# Matrix Gla protein maintains normal and malignant hematopoietic progenitor cells by interacting with bone morphogenetic protein-4

**DOI:** 10.1016/j.heliyon.2020.e03743

**Published:** 2020-04-12

**Authors:** Kana Kuronuma, Aya Yokoi, Tomoya Fukuoka, Muneaki Miyata, Akio Maekawa, Satowa Tanaka, Leo Matsubara, Chie Goto, Miki Matsuo, Hao-Wei Han, Mai Tsuruta, Haruka Murata, Hikari Okamoto, Natsumi Hasegawa, Shigetaka Asano, Mitsuhiro Ito

**Affiliations:** a Laboratory of Hematology, Division of Medical Biophysics, Kobe University Graduate School of Health Sciences, 7-10-2 Tomogaoka, Suma-ku, Kobe 654-0142, Japan; b Division of Pathogenetic Signaling, Department of Biochemistry and Molecular Biology, Kobe University Graduate School of Medicine, CREST, Japan Science and Technology Agency, 1-5-6 Minatojima-minamimachi, Chuo-ku, Kobe, 650-0047, Japan; c Research Organization for Nano & Life Innovation, Waseda University, 3-4-1 Okubo, Shinjuku-ku, Tokyo 159-8555, Japan

**Keywords:** Biochemistry, Cancer research, Cell biology, Molecular biology, Physiology, Stem cells research, Biomolecules, Molecular dynamics, Hematological system, Oncology, Matrix Gla protein (MGP), Bone morphogenetic protein-4 (BMP-4), BMP-2, Hematopoietic niche, Mediator transcriptional coregulatory complex

## Abstract

Matrix Gla protein (MGP), a modulator of the BMP-SMAD signals, inhibits arterial calcification in a Glu γ-carboxylation dependent manner but the role of MGP highly expressed in a subset of bone marrow (BM) mesenchymal stem/stromal cells is unknown. Here we provide evidence that MGP might be a niche factor for both normal and malignant myelopoiesis. When mouse BM hematopoietic cells were cocultured with mitomycin C-treated BM stromal cells in the presence of anti-MGP antibody, growth of hematopoietic cells was reduced by half, and maintenance of long-term culture-initiating cells (LTC-ICs) was profoundly attenuated. Antibody-mediated blockage of MGP also inhibited growth (by a fifth) and cobblestone formation (by half) of stroma-dependent MB-1 myeloblastoma cells. MGP was undetectable in normal hematopoietic cells but was expressed in various mesenchymal cells and was aberrantly high in MB-1 cells. MGP and bone morphogenetic protein (BMP)-4 were co-induced in stromal cells cocultured with both normal hematopoietic cells and MB-1 myeloblastoma cells in an oscillating several days-periodic manner. BMP-2 was also induced in stromal cells cocultured with normal hematopoietic cells but was barely expressed when cocultured with MB-1 cells. GST-pulldown and luciferase reporter assays showed that uncarboxylated MGP interacted with BMP-4 and that anti-MGP antibody abolished this interaction. LDN-193189, a selective BMP signaling inhibitor, inhibited growth and cobblestone formation of MB-1 cells. The addition of warfarin, a selective inhibitor of vitamin K-dependent Glu γ-carboxylation, did not affect MB-1 cell growth, suggesting that uncarboxylated MGP has a biological effect in niche. These results indicate that MGP may maintain normal and malignant hematopoietic progenitor cells, possibly by modulating BMP signals independently of Glu γ-carboxylation. Aberrant MGP by leukemic cells and selective induction of BMP-4 relative to BMP-2 in stromal cells might specify malignant niche.

## 1 Introduction

Hematopoiesis is a lifelong and continuous replenishment of hematopoietic stem cells (HSCs) and their differentiation into multi-lineage mature progenies. These processes are induced by intrinsic and extrinsic factors, the latter of which are molecules produced by a hematopoietic microenvironment called a niche that resides perivascular [1, 2]. Mesenchymal stromal cells comprise the major niche components and are sources for extrinsic niche molecules [2, 3]. Among the TGF-β family proteins, bone morphogenetic protein (BMP)-4 increases HSCs through SMAD signals during development, while TGF-β inhibits their growth [4]. Neoplastic myeloid (or leukemic) stem/initiating cells (LSCs) also depend on the niche, and may share the normal niche molecules but may modify the niche to suit themselves. Alternatively, an altered niche may at times precede the onset of hematopoietic neoplasms. In such situations, neoplastic cells may use distinct niche molecules that these cells favor rather than normal blood cells [5, 6].

The general transcriptional coregulatory complex Mediator, a subcomplex of the RNA polymerase II holoenzyme, is the endpoint convergence of a variety of intracellular signals, and initiates mRNA transcription [7, 8, 9]. Among the circa 31 Mediator subunits, MED1 is a specific coactivator for activators including nuclear receptors [7] and is crucial for transcription of genes involved in hematopoietic niche function [10]. We previously identified dozens of genes whose expressions were significantly attenuated in *Med1*^−/−^ mesenchymal stromal cells [10], and reported that osteopontin, fibroblast growth factor 7 (FGF7) and periostin independently supported hematopoietic stem or progenitor cells (HSPCs) *in vitro* [10, 11, 12].

An 85-residue 10-kDa protein, matrix Gla protein (MGP), which was originally identified as a γ-carboxyglutamic acid (Gla)-containing protein that was associated with the bovine bone matrix [13, 14], is now highlighted in a context of molecular taxonomy of BM stroma, as it is abundantly expressed specifically in a subset of bone marrow (BM) leptin-receptor-positive mesenchymal stem/stromal cells, major components of the BM hematopoietic microenvironment, and their descendent osteolineage cells [15, 16]. MGP reportedly interacts with BMP-4 and BMP-2 and modulates the BMP-SMAD signals [17, 18]. The *MGP* promoter has putative binding sites for vitamin D and retinoic acid receptors [14], and vitamin D enhances MGP expression in bone cells [19], indicating *MGP* as a putative MED1-targeted gene. MGP is known as a functional inhibitor of calcification: MGP-deficient mice die of arterial ectopic calcification associated with activated BMP signals and subsequent rupture [20, 21]; and patients with Keutel syndrome, whose MGP is nonfunctional, suffer from diffuse cartilage calcification and mid-facial dysmorphism [22]. The inhibition of ossification appears to depend on the Glu γ-carboxylation of MGP, as uncarboxylated MGP is associated with arterial stiffness in humans [23]. Apart from the role for MGP in inhibiting calcification, however, biological action of MGP expressed abundantly in BM stromal cells has been veiled. Recently, MGP has been identified as a metastasis-related poor-prognostic factor for osteosarcoma, and notably, its prometastatic activity is independent of Glu γ-carboxylation [24], indicating that uncarboxylated MGP is functional in a setting other than ossification.

In this study, we looked at MGP whose expression was profoundly attenuated in *Med1*^−/−^ mesenchymal stromal cells and MGP-interacting proteins, and aimed at evaluating if MGP had a niche function. We propose that MGP may be a novel niche factor for both normal and malignant HSPCs.

## 2 Materials and methods

### 2.1 Cell culture

The MS-5 cells [25], OP-9 cells, distributed by RIKEN BRC through the National Bio-Resource of the Ministry of Education, Culture, Sports, Science and Technology of Japan (MEXT), and MC3T3-E1 cells, were maintained as described [10]. Mouse BM-derived primary mesenchymal stem/stromal cells (MSCs; Gibco), and *Med1*^+/+^ and *Med1*^−/−^ mouse embryonic fibroblasts (MEFs) that harbored a p53 inactivation [10], were cultured in Dulbecco's modified Eagle's medium (DMEM) supplemented with 10% fetal bovine serum (FBS) [10]. The MB-1 niche-dependent myeloblastoma cells [26, 27] were maintained by coculturing with mitomycin C (MMC)-treated OP-9 cells and used by coculturing with MMC-treated either MS-5 or OP-9 cells [12]. In some experiments, 0.5 μM LDN-193189 (Selleck) or 10 μM warfarin (Wako) was added to the coculture [24].

### 2.2 BM hematopoietic cell culture

BM hematopoietic cells were prepared from femora and tibiae of C57BL6 mice [12]. These cells (5 × 10^5^ cells/well) were added to 24-well plates in which MMC-treated MS-5 or OP-9 cells, or MEFs, were pre-seeded, and cultured in Methocult M5300 (STEMCELL Technologies) in the absence or presence of 0.5 mg/ml anti-mouse (m) MGP goat polyclonal antibody (sc-32820; Santa Cruz Biotechnology) or normal goat IgG (R&D Systems) [10]. In some experiments, Transwell plates (12-well, Corning) were used to analyze the effect of cell adhesion between hematopoietic cells and stromal cells.

For long-term culture-initiating cells (LTC-ICs), BM hematopoietic cells cocultured with MMC-treated MS-5 or OP-9 stromal cells for 4 weeks were placed in complete methylcellulose medium (Methocult M3434; STEMCELL Technologies) for 2 weeks, and colonies were counted [10, 12].

All animal experiments were performed according to the institutional guidelines of the Animal Research Center, Kobe University, Japan, after the ethical approval from the Kobe University Animal Experiments Committee.

### 2.3 DNA synthesis

DNA synthesis was measured through the incorporation of bromodeoxyuridine (BrdU). After treating cells with 100 μM BrdU (Roche) for 24 h, its incorporation into DNA was measured using the Cell Proliferation ELISA, BrdU (colorimetric) (Roche) [10].

### 2.4 Quantitative RT-PCR

Total RNA (0.5 μg), extracted using Isogen II (Nippon Gene), was reverse-transcribed using the ReverTra Ace qPCR RT Master Mix with gDNA Remover (Toyobo). Then quantitative PCR (StepOnePlus Real-Time PCR system; Thermo Fisher) was performed to measure mRNA levels. Values of mRNA for glyceraldehyde-3-phosphate dehydrogenase (GAPDH) were used to normalize the results. The sequences of the primers used in amplification are available upon request.

### 2.5 Western blot and ELISA

For western blotting, total cell lysates were separated through SDS-PAGE, transferred to nitrocellulose membranes and probed with antibodies against mMGP and β-actin (MAB1501; Merck Millipore). Chemiluminescence was detected by an ImageQuant LAS 4000mini (GE Healthcare).

For quantification of mMGP in media, ELISA was performed by using ELISA Kit for Matrix Gla Protein (SEB477Mu; Cloud-Clone).

### 2.6 Glutathione S-transferase (GST)-pulldown assay

The cDNA encoding mMGP (20–104) without the signal peptide was amplified through PCR using the primer pair 5ʹ-GCGGATCCTACGAATCTCACGAAAGCATGGAG-3ʹ and 5ʹ-CGGAATTCTAATATTTGGCTCCTCGGCGCTG-3ʹ and was cloned in pGEX4T1 (GE Healthcare) after restriction digestion with BamHI and EcoRI. Recombinant GST-mMGP and GST were bacterially expressed.

For *in vitro* protein–protein interaction analysis, immobilized GST or GST-mMGP (10 μg) and recombinant mBMP-4 or mBMP-2 (Wako) (50 ng) were incubated in BC150 buffer with 0.1% NP-40 and 1 mM β-mercaptoethanol at 4 °C for 1 h. The beads were then washed extensively with the binding buffer. Bound proteins were eluted in 0.3% sarkosyl and detected through western blotting.

### 2.7 Mammalian two-hybrid assay

The cDNA encoding mMGP (20–104) was fused to GAL4 and subcloned into pCDM8 (Invitrogen) to generate pGal4-mMGP. The cDNAs encoding secreted forms of mBMP-4 and mBMP-2 (mBMP-4 (293–408) and mBMP-2 (281–394)), were fused to the VP16 activation domain and subcloned into pcDNA3.1neo (Thermo Fisher) to create pVP16-mBMP-4 and pVP16-mBMP-2.

For mammalian two-hybrid assays, cells (2 × 10^4^) in 24-well plates were transfected with pGal4-mMGP (10 ng) and either pVP16-mBMP-4 or pVP16-mBMP-2 (150 ng), together with 5 × GAL4-LUC (100 ng) and the *Renilla* control luciferase vector (5 ng) using Lipofectamine 2000 (Thermo Fisher).

### 2.8 Statistical analyses

Results (N = 4, if unspecified otherwise) were shown as means ± SD, and analyzed using Student's *t*-test. Two-way ANOVA was used for studies conducted over a prolonged period and, when significant, values for each time point were evaluated using Student's *t*-test. P < 0.05 and P < 0.01 were represented by ∗ and ∗∗, respectively.

## 3 Results

### 3.1 Antibody-mediated blockage of MGP reduces proliferation of cocultured BM hematopoietic cells and HSPCs support

In the view that MED1 in BM stromal cells is necessary for optimal hematopoietic cell growth and HSPC support, that transcription of vitamin D-targeted *Mgp* is reduced in MED1-deficient stromal cells [10], and that a subset of BM stromal cells highly expresses MGP [15, 16], we hypothesized that MGP may be a novel niche factor for hematopoiesis. To explore the aptness of this hypothesis, we analyzed the effect of downregulation of MGP produced by BM stromal cells in *in vitro* hematopoietic coculture.

Repeated endeavors of CRISPR-Cas9-mediated *Mgp* inactivation in MS-5 stromal cells resulted in failure, after screening clones derived from 288 GFP-positive single cells each for three different targeting vectors, indicating that MS-5 cells might have required MGP for survival/proliferation (supplementary Figure 1). Hence, we performed experiments by using the blocking antibody against mMGP.

**Figure 1:**
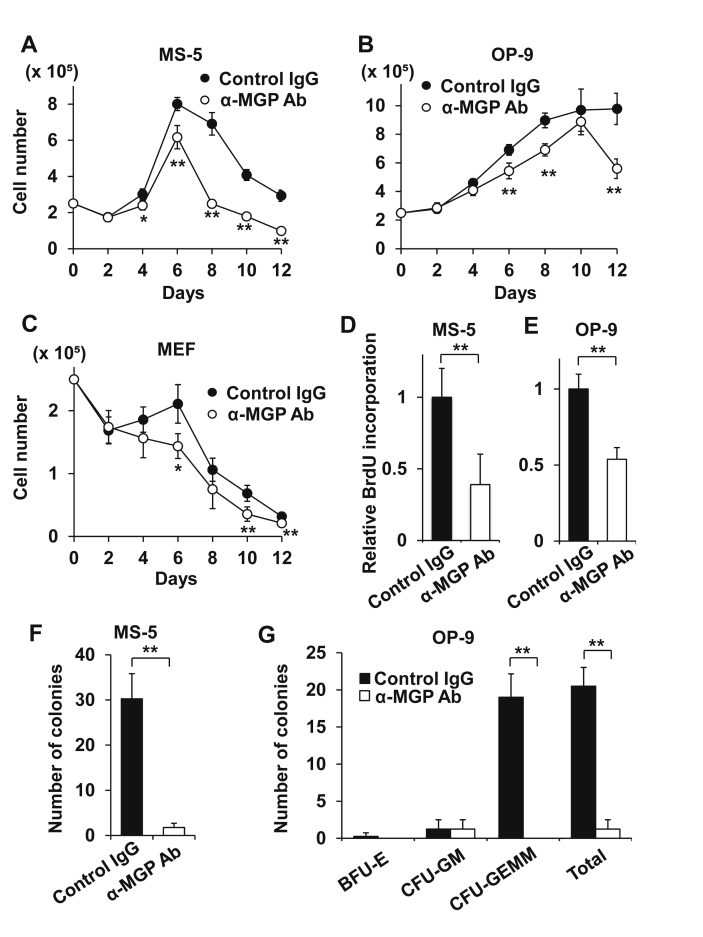
Antibody-mediated blockage of MGP attenuates growth and support of normal BM hematopoietic cells cocultured with MS-5 or OP-9 BM stromal cells. (A,B,C) Growth of normal BM hematopoietic cells, cocultured with MS-5 (A) or OP-9 (B) cells, or MEFs (C) was attenuated in the presence of anti-MGP antibody. (D,E) DNA synthesis (day 4) of normal BM hematopoietic cells cocultured with MS-5 (D) or OP-9 (E) cells were attenuated in the presence of anti-MGP antibody. (F,G) LTC-IC-derived colonies after coculture with MS-5 (F) or OP-9 (G) cells were attenuated in the presence of anti-MGP antibody. CFU-GEMM (corresponding to common myeloid progenitors) were particularly lost by the antibody. The result of LTC-ICs, without limiting dilution, is qualitative rather than quantitative.

When BM hematopoietic cells were cocultured with MS-5 or OP-9 BM stromal cells or MEFs in the presence of anti-MGP antibody for 12 days, the number of hematopoietic cells was attenuated by over 20% compared to the controls after four days of coculture (Figure 1A-C). However, the number of dead cells counted through trypan blue staining was comparable in both groups, indicating that cell death was unaffected. Antibody addition also reduced DNA synthesis by half in hematopoietic cells after coculturing with MS-5 or OP-9 cells (Figure 1D,E).

We next explored the effect of the antibody in support of HSPCs. When BM hematopoietic cells were cocultured with MS-5 or OP-9 cells in the presence of anti-MGP antibody for 4 weeks, the number of colonies derived from LTC-ICs, particularly common myeloid progenitors (CFU-GEMM), was profoundly reduced compared to the controls (Figure 1F,G). These results collectively suggest that MGP has a role in enhancing hematopoietic cell growth and HSPCs support.

### 3.2 Antibody-mediated blockage of MGP reduces growth/maintenance of MB-1 myeloblastoma cells

**Figure 2:**
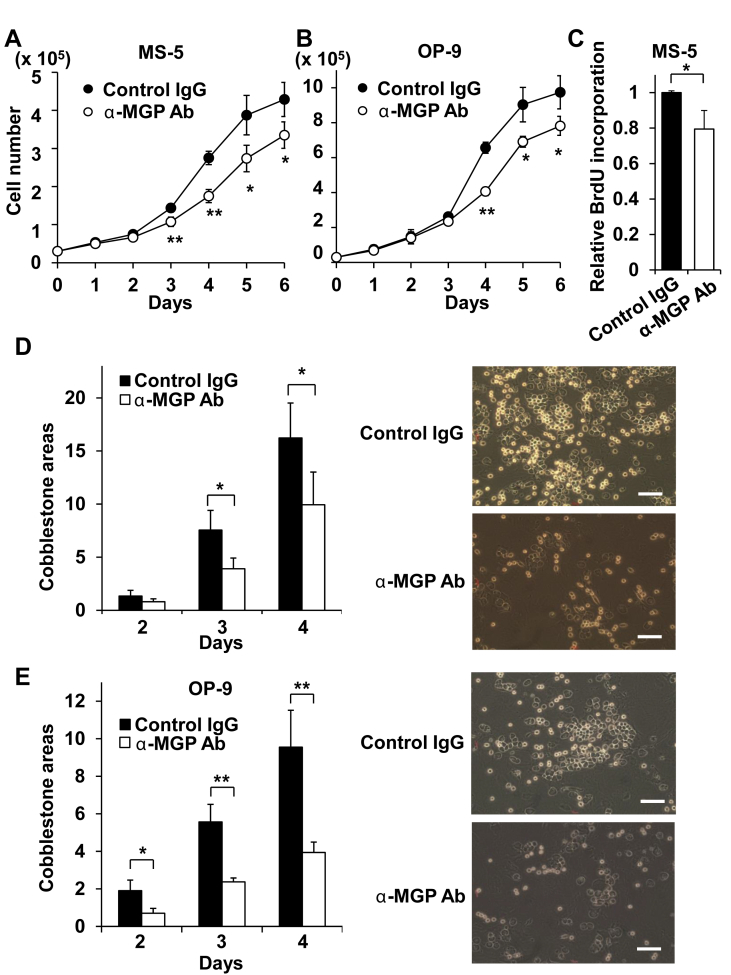
Antibody-mediated blockage of MGP attenuates growth and support of MB-1 myeloblastoma cells cocultured with MS-5 or OP-9 BM stromal cells. (A,B) Growth of MB-1 myeloblastoma cells cocultured with MS-5 (A) or OP-9 (B) cells was attenuated in the presence of anti-MGP antibody. (C) DNA synthesis (day 4, N = 3) of MB-1 myeloblastoma cells was attenuated in the presence of anti-MGP antibody. (D,E) Cobblestone areas of MB-1 myeloblastoma cells cocultured with MS-5 (D) or OP-9 (E) cells were attenuated in the presence of anti-MGP antibody. Bar, 100 μm.

Niche-dependent MB-1 myeloblastoma cells, established from a patient with blast crisis chronic myeloid leukemia, require stromal cells for survival and growth [26, 27]. These cells grow by forming cobblestone areas beneath stromal cells, a characteristic feature of LSCs, but die of apoptosis when dissociated from stromal cells [27]. We next utilized MB-1 cells as a model to analyze the effect of MGP in the growth/maintenance of malignant hematopoietic precursors. When MB-1 cells were cocultured with MS-5 or OP-9 cells in the presence of anti-MGP antibody, the number of MB-1 cells was attenuated by over 20% compared to the controls (Figure 2A,B), although the number of dead cells was comparable in both groups (less than 5%). The antibody addition also reduced DNA synthesis by a fifth upon coculturing MB-1 cells with MS-5 cells (Figure 2C). Of note, the antibody profoundly reduced the number of cobblestone areas in cocultures with both MS-5 and OP-9 cells by 40% and 50%, respectively, after 2 days (Figure 2D,E). These results suggest that MGP promotes the growth/maintenance of MB-1 myeloblastoma cells.

### 3.3 MGP is abundantly produced by mesenchymal cells but not by normal hematopoietic cells

**Figure 3:**
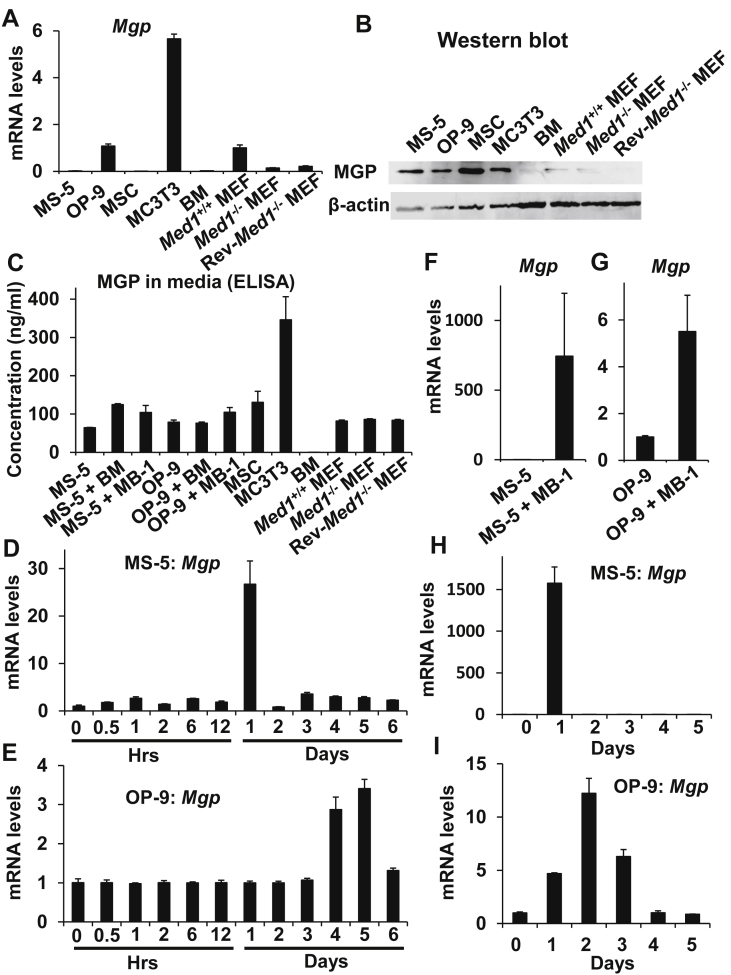
Coculture with BM hematopoietic cells or MB-1 cells induces *Mgp* in MS-5 or OP-9 cells. (A,B) *Mgp* mRNA (A) and MGP (B) were variably expressed in various mesenchymal cells and BM hematopoietic cells and attenuated in *Med1*^−/−^ MEFs. The full non-adjusted images of the blots are provided in Supplementary Material (B). (C) MGP was secreted from mesenchymal cells but not from BM hematopoietic cells. (D,E) *Mgp* was induced in MS-5 (D) or OP-9 (E) cells during coculture with normal BM hematopoietic cells. (F,G) *Mgp* was induced in MS-5 (F) or OP-9 (G) cells during coculture with MB-1 myeloblastoma cells. (H,I) *Mgp* was induced in MS-5 (H) or OP-9 (I) cells when cocultured with dissociated BM hematopoietic cells in Transwell plates.

*Mgp* mRNA was variably expressed in various mesenchymal cells and BM hematopoietic cells. *Med1*^−/−^ MEFs transcribed only 14% of *Mgp* that was transcribed by *Med1*^+/+^ MEFs; the recovery of *Mgp* transcription in the revertant *Med1*^−/−^ MEFs in which MED1 was reintroduced (Rev-*Med1*^−/−^ MEFs) was low (1.5-fold), indicating that the contribution of vitamin D (or other nuclear hormone signals) was limited and that the role of MED1 was rather indirect (Figure 3A). Intracellular MGP levels were also variable in these cells (Figure 3B). Notably, MGP secreted through its signal peptide into the media was high in various mesenchymal cells, but was barely detected in BM hematopoietic cells (Figure 3C). Consistently with these data, single cell RNAseq of human and mouse BM hematopoietic stem and various precursor cells deposited in the public database showed hardly any transcription of genes encoding MGP, if not none [28, 29], indicating a majority of MGP in normal hematopoietic niche is produced by BM stromal cells. As niche action is attributed to secreted MGP, the anti-MGP antibody activity (above) is thought to mainly be directed towards MGP produced by stromal cells.

### 3.4 Stromal cell Mgp is induced during coculture with hematopoietic cells

During the coculture of normal BM hematopoietic cells with MS-5 or OP-9 BM stromal cells, *Mgp* was induced prominently (up to 27- or 3-fold) and transiently after one day (Figure 3D,E). *Mgp* transcription in MS-5 or OP-9 was also prominently enhanced (744- or 6-fold) when cocultured with MB-1 myeloblastoma cells (Figure 3F,G). *Mgp*, transcribed in OP-9 or MS-5 stromal cells, was likewise transiently induced (1577- or 12-fold) after one day of coculture with BM hematopoietic cells that were physically dissociated from stromal cells by the Transwell apparatus (Figure 3H,I), indicating that secreted humoral factor(s) provoked *Mgp* expression in stromal cells for an action as a niche factor.

### 3.5 Stromal cell Bmp-4 and Bmp-2 are induced during coculture with BM hematopoietic cells

**Figure 4:**
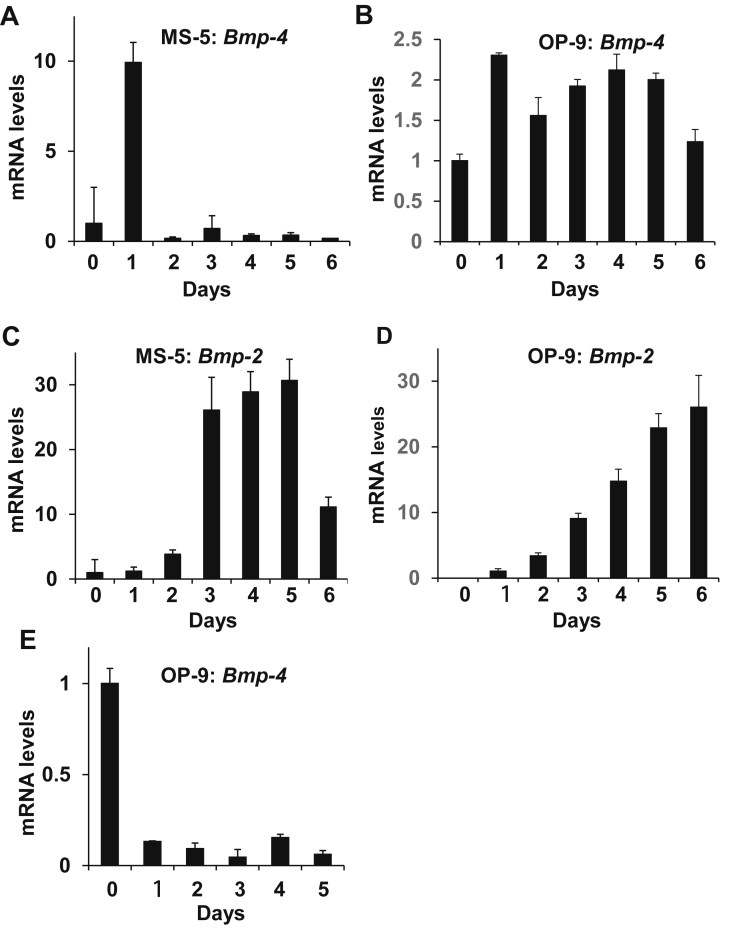
Coculture with BM hematopoietic cells induces *Bmp-4* and *Bmp-2* in MS-5 or OP-9 cells. (A–D) *Bmp-4* was induced in MS-5 (A,B) or OP-9 (C,D) cells simultaneously with *Mgp* (A,C), and *Bmp-2* was later induced (B,D), during coculture with BM hematopoietic cells. (E) *Bmp-4* was suppressed in OP-9 cells when cocultured with dissociated BM hematopoietic cells in Transwell plates.

Since the support of HSPCs requires their association with stromal cells, induced MGP expression was apparently insufficient *per se* but acted indirectly for HSPCs support. Therefore, we next asked if BMP-4 and BMP-2, which reportedly associate with MGP, were also induced upon coculture and were employed in HSPCs support jointly with MGP. Indeed, during coculture with MS-5 or OP-9 cells, *Bmp-4* was rapidly and transiently induced (10- or 2-fold) within a few days (Figure 4A,B), simultaneously with MGP, followed by subsequent and sustained induction of *Bmp-2* (31- or 26-fold) that lasted over a week (Figure 4C,D). However, neither *Bmp-4* nor *Bmp-2* was induced by MS-5 or OP-9 cells when cultured with dissociated BM hematopoietic cells using the Transwell apparatus: transcription of *Bmp-4*/*Bmp-2* in MS-5 cells and *Bmp-2* in OP-9 cells was undetected, and solely *Bmp-4* transcription was detected weakly (less than 20%) (Figure 4E). Therefore, *Bmp-4*/*Bmp-2* induction depended on the physical association between hematopoietic cells and stromal cells. The inductions of *Bmp-4/Bmp-2* and *Mgp* were most probably regulated differentially, as the former was dependent, and the latter independent, of the interaction between hematopoietic cells and stromal cells.

### 3.6 Stromal cell Bmp-4, but not Bmp-2, is induced during coculture with MB-1 myeloblastoma cells

**Figure 5:**
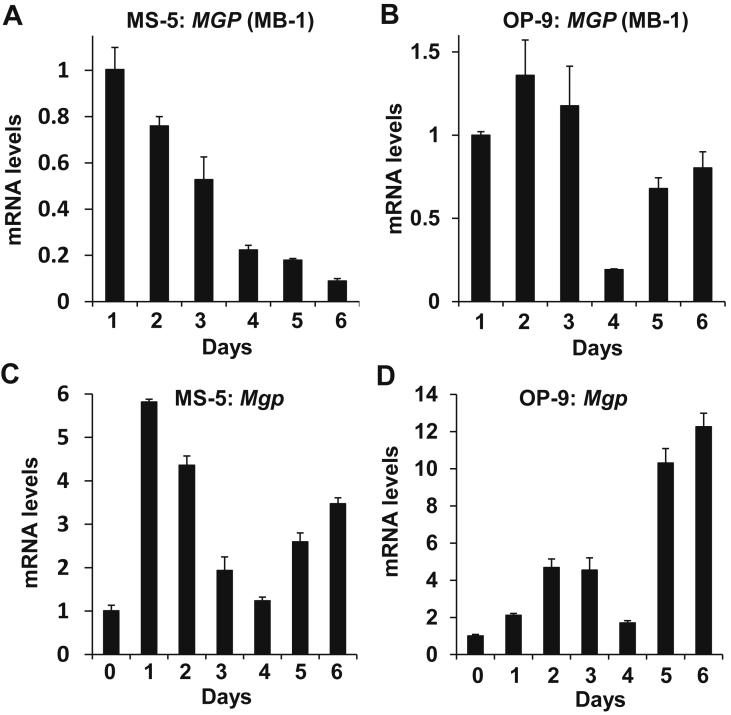
MB-1 cells express *MGP* ectopically and induce *Mgp* in cocultured MS-5 or OP-9 stromal cells. (A,B) *MGP* is ectopically expressed in an oscillated manner in MB-1 cells cocultured with MS-5 (A) or OP-9 (B) cells. (C,D) *Mgp* in MS-5 (C) or OP-9 (D) cells cocultured with MB-1 cells were periodically expressed.

In contrast to a scarce production of MGP by normal hematopoietic cells (above), MB-1 myeloblastoma cells (presumably ectopically) transcribed a meaningfully high level of *MGP* that was comparable to the *GAPDH* level (Figure 5A,B). *MGP* expression in MB-1 cells was accompanied by an oscillating several days-periodic induction (up to 6- or 12-fold) of *Mgp* in MS-5 or OP-9 stromal cells (Figure 5C,D).

**Figure 6:**
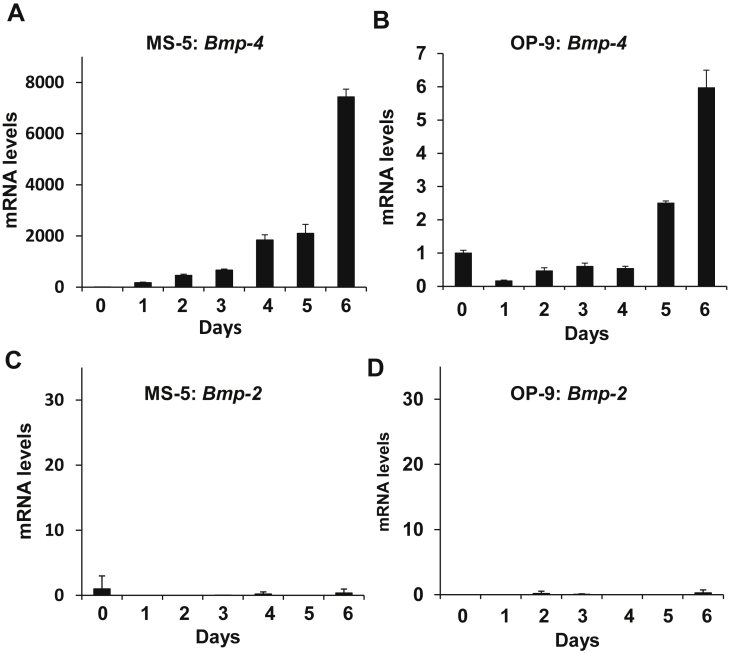
Coculture of MB-1 myeloblastoma cells induces *Bmp-4*, but not *Bmp-2* in MS-5 or OP-9 cells. (A–D) *Bmp-4* was highly induced in MS-5 (A) or OP-9 (B) cells, while *Bmp-2* remained suppressed in MS-5 (C) or OP-9 (D) cells.

During coculture with MB-1 cells, *Bmp-4* began to be induced after one day, and the transcription reached 7434- and 6-fold increases after 6 days, in both MS-5 and OP-9 cells (Figure 6A,B). However, in contrast to the situation of stromal cells cocultured with normal hematopoietic cells, *Bmp-2* was not induced (Figure 6C,D). Differential *Bmp-4* and *Bmp-2* transcription in stromal cells, together with ectopically high *MGP* transcription in MB-1 cells (above), might specify a malignant niche.

### 3.7 Uncarboxylated mMGP interacts with BMP-4 and BMP-2

**Figure 7:**
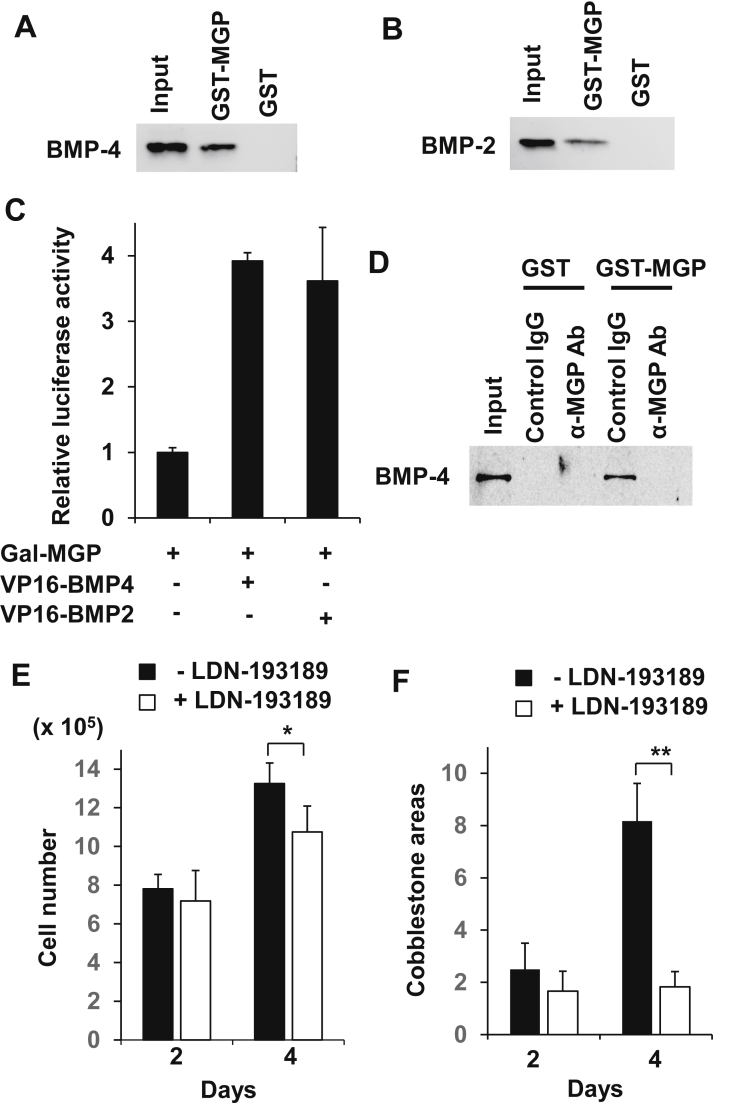
Uncarboxylated MGP interacts with BMP-4 and inhibition of BMP signaling suppresses growth/maintenance of MB-1 cells cocultured with MS-5 cells. (A,B,C) GST-pulldown (A,B) and luciferase reporter (C) assays showed interaction between mMGP and mBMP-4/mBMP-2. (D) Anti-MGP antibody blocked interaction between mMGP and mBMP-4. The full non-adjusted images of the blots are provided in Supplementary Material (A,B,D). (E,F) Growth (E) and cobblestone formation (F) of MB-1 cells cocultured with MS-5 cells were attenuated in the presence of LDN-193189. N = 4 (E) or 3 (F).

The simultaneous induction of MGP and BMP-4 in stromal cells during coculture with both normal and malignant hematopoietic cells, and previous reports demonstrating that γ-carboxylated MGP interacts with BMP-4/BMP-2 [17,18], prompted us to ask if MGP acted by interacting with BMP-4 in our coculture system. Somewhat surprisingly, recombinant GST-mMGP, which in bacteria was not γ-carboxylated at the Glu residues, physically associated with both recombinant mBMP-4 and mBMP-2 (Figure 7A,B). Mammalian two-hybrid assays also showed specific interactions between mMGP (which was assumed to be uncarboxylated intracellularly) and mBMP-4/mBMP-2 (Figure 7C). These results suggest that uncarboxylated mMGP interacts with mBMP-4/mBMP-2.

Since *Bmp-4* was induced simultaneously with *Mgp* but *Bmp-2* was not in stromal cells associated with MB-1 cells, we analyzed the effect of the anti-MGP antibody in the MGP−BMP-4 interaction. When recombinant GST-mMGP and mBMP-4 were incubated together with the anti-MGP antibody at the same molar ratio as GST-mMGP, the interaction between mMGP and mBMP-4 was abolished (Figure 7D). This result indicates that the antibody inhibits the MGP−BMP-4 interaction, which may be the mechanism underlying deterred hematopoietic cell growth and support.

### 3.8 Inhibition of BMP signal downregulates growth/support of MB-1 myeloblastoma cells

As the antibody apparently affected the BMP signal through inhibiting the MGP−BMP-4 interaction, we analyzed the effects of inhibiting BMP signaling in MB-1 cells. LDN-193189 is a potent and selective inhibitor of the BMP type I receptors ALK2 and ALK3 and inhibits BMP4-mediated Smad1/5/8 activation [30]. Indeed, when MB-1 cells were cocultured with MS-5 cells in the presence of LDN-193189, growth of MB-1 cells was retarded by a fifth compared to the control after 4 days (Figure 7E). Cobblestone formation of MB-1 cells was simultaneously attenuated in the presence of LDN-193189, being a quarter of the control, after 4 days (Figure 7F). These results support the abovementioned idea that MGP promotes growth and maintenance of MB-1 cells through interacting with BMP-4.

### 3.9 Inhibition of Glu γ-carboxylation does not affect MB-1 myeloblastoma cell growth/support

The fact that uncarboxylated MGP interacted with BMPs and that the interaction was inhibited by anti-MGP antibody led us to hypothesize that uncarboxylated MGP is a physiologically active niche molecule. Glu γ-carboxylation is dependent on an active form of vitamin K catalyzed by the vitamin K epoxide reductase complex subunit 1 (VKORC1). Warfarin, vitamin K antagonist, is a highly potent and selective inhibitor for VKORC1, and has long been used in clinic as an anticoagulant [31]. Warfarin activity is so specific that off-target pharmacological effects unrelated to vitamin K-antagonizing effects have not been described [32]. Notably, side effects of warfarin include non-uremic calciphylaxis: it involves vascular calcification that occurs after a prolonged administration of warfarin and is suggested to be attributed to uncarboxylated MGP [33].

**Figure 8:**
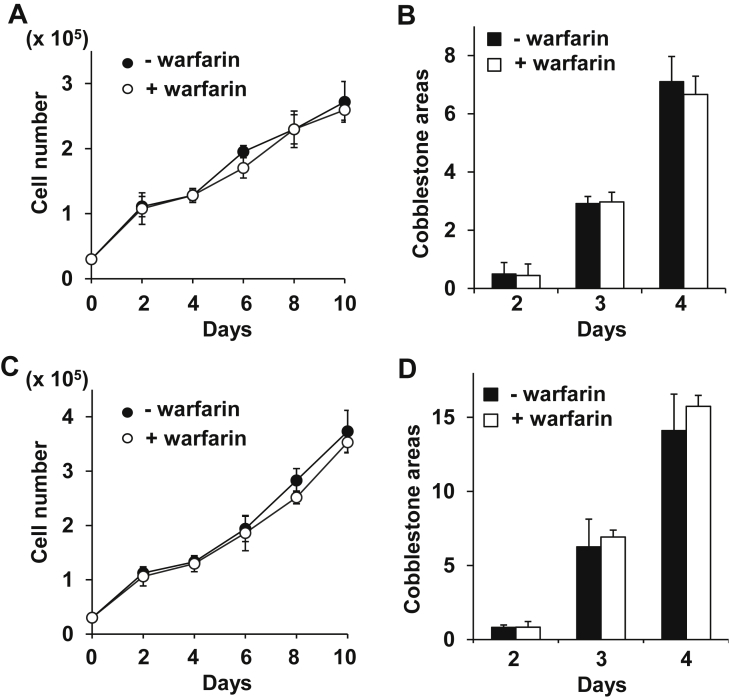
Uncarboxylated MGP supports growth/maintenance of MB-1 cells cocultured with MS-5 or OP-9 cells. (A–D) Warfarin did not affect growth (A,C) and cobblestone areas (B,D) of MB-1 cells cocultured with MS-5 (A,B) or OP-9 (B,D) cells.

We, thus, added warfarin to the coculture of MB-1 myeloblastoma cells and MS-5 or OP-9 stromal cells to block vitamin K action in these cells. Indeed, warfarin addition did not affect MB-1 cell growth and formation of cobblestone areas (Figure 8A-D). These results may suggest that uncarboxylated MGP is an active niche molecule.

## 4 Discussion

We propose MGP as a novel niche factor for normal and malignant hematopoietic progenitors. MGP appears to act in part through interacting with the BMP signals. The role for MGP in the myeloblastic niche is particularly highlighted with regard to specific induction of BMP-4 in stromal cells and ectopic expression of MGP in myeloblastoma cells. In contrast to the role of MGP in inhibiting calcification, the niche action of MGP does not depend on its Glu γ-carboxylation. This study is the first report that signifies a role of MGP on hematopoiesis.

Stromal cells do not support dissociated hematopoietic cells [10], even if the latter cells induce *Mgp* in remote stromal cells (this study). Hence MGP does not act as a niche factor alone but needs (an)other molecules for the action. Our observations suggest that the MGP activity is coupled with a physical interaction with BMP and subsequent BMP signaling, leading to a model that posits: (1) physical interaction between hematopoietic precursor cells and stromal cells; (2) oscillated induction by stromal cells of genes encoding MGP and BMP-4/2; (3) association of MGP with BMP-4/2; and (4) induction of BMP signaling through BMP type I receptors.

Our study also leads to a provisional supposition that malignant hematopoietic niche might be specified by (1) aberrant MGP copiously produced by leukemic cells, and (2) selective induction of BMP-4 relative to BMP-2 by stromal cells. Although underlying mechanisms are currently unknown, it is possible that the resultant abnormal niche in turn causes the production of malignant hematopoietic progenitor cells.

A precedent to the role for MGP in possible niche function has been documented with regard to osteosarcoma metastasis. Ectopic MGP expression in osteosarcoma cells promotes lung metastasis independently of Glu γ-carboxylation of MGP [24]. Thus, MGP appears to enhance the ectopic homing of osteosarcoma cells in lungs, where, as a secreted bioactive molecule, it may act on lung parenchyma to induce factor(s) for the survival/growth of osteosarcoma cells. Together with our finding in niche model that MGP may constitute a specific malignant microenvironment concomitant with the induction of BMP-4 in mesenchymal cells, MGP might be regarded more broadly as a niche factor that modifies microenvironments to suit cancer stem cells for survival and growth. This function of MGP appears to be independent of Glu γ-carboxylation, indicating that there might be hitherto unrecognized mechanism(s) of action that MGP employs.

MGP also interacts with some extracellular matrix proteins that include elastin, fibronectin, and vitronectin [34, 35, 36]. These interactions, and subsequent sequelae of modulated intercellular communications, may also contribute to the unidentified function of MGP as a niche molecule, and should be studied in detail in the future.

MGP reportedly acts on BMP signaling in a multiphasic manner: low and high levels of MGP relative to BMP-2 promote the osteoinductive effect of BMP-2, while intermediate levels of MGP strongly inhibit it [18]. Our study that hints the action of uncarboxylated MGP on BMP signaling adds to the complexity of the multifaceted roles of MGP. Simplified *in vitro* models using biochemistry or cell biology (as in this study) would be useful to elucidate the mechanisms. However, the limitation of these functional studies based on the use of the polyclonal antibody against MGP should also be noted, and similar assays with a genetic approach are desired in future. And once MGP actions are modeled *in vitro*, it would be worth analyzing the *in vivo* relevance (in mice) to define MGP physiology.

## Declarations

### Author contribution statement

M. Miyata: Conceived and designed the experiments; Performed the experiments; Analyzed and interpreted the data; Contributed reagents, materials, analysis tools or data.

L. Masubara and H. Han: Conceived and designed the experiments; Performed the experiments; Analyzed and interpreted the data.

N. Hasegawa: Conceived and designed the experiments; Analyzed and interpreted the data.

S. Asano: Conceived and designed the experiments; Analyzed and interpreted the data; Contributed reagents, materials, analysis tools or data.

M. Ito: Conceived and designed the experiments; Analyzed and interpreted the data; Contributed reagents, materials, analysis tools or data; Wrote the paper.

K. Kuronuma, A. Yokoi and T. Fukuoka: Performed the experiments; Analyzed and interpreted the data.

A. Maekawa, S. Tanaka, C. Goto, M. Matsuo, M. Tsuruta, H. Murata and H. Okamoto: Performed the experiments.

### Funding statement

This work was supported by Grants-in-Aid for Scientific Research (26460677, 17K09012 and 20K07782) from the Japan Society for the Promotion of Science, and by the research fund from Chugai Pharmaceutical Co. Ltd. to Waseda University.

### Competing interest statement

The authors declare no conflict of interest.

### Additional information

No additional information is available for this paper.
