# Supplementary material for: Matrix Gla protein maintains normal and malignant hematopoietic progenitor cells by interacting with bone morphogenetic protein-4
Source: Heliyon. 2020 Apr 12;6(4):e03743. doi: 10.1016/j.heliyon.2020.e03743 (PMC7160454; doi:10.1016/j.heliyon.2020.e03743)
Supplement: Supplementary Figure 1 [file mmc1.pdf]

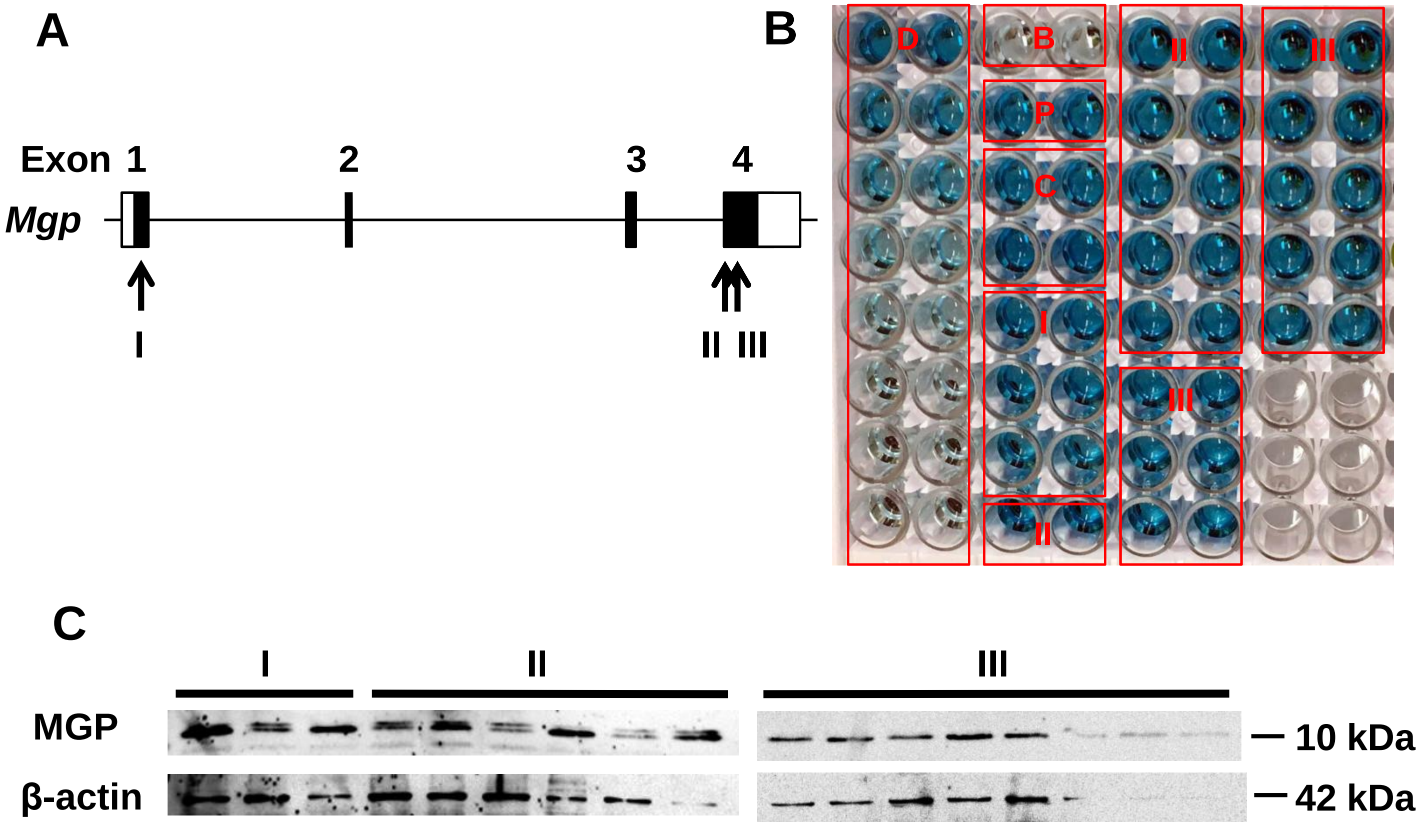

Supplementary Fig. 1. MS-5 cells, whose *Mgp* was inactivated, were unobtainable.

(A) Schematic representation of the targeting sites of guide (g) RNAs, I (within exon 1), II and III (within exon 4), for the CRISPR-Cas9 system disrupting the *Mgp*. Three targeting vectors containing the gRNA sequences, and an empty vector, were each introduced into MS-5 cells. Among 96 single cells into which each of these vectors was introduced, cells in 3 (vector I), 5 (vector II), 8 (vector III), and 50 (empty control vector) wells grew and formed clones.

(B) ELISA. MGP concentrations in culture media of the obtained cells, as well as the parental cells, were measured. All of the obtained clones produced immunoreactive mMGP. D, dilution series of recombinant mMGP for the standardization; B, blank; P, parental cells; C, control empty vector; I, vector I; II, vector II; and III, vector III.

(C) Western blot analysis. All of the clones contained full-length mMGP, indicating that *Mgp* was not knocked out in MS-5 cells. The full non-adjusted images of the blots are provided in supplementary material.

Method. Three gRNA sequences were designed through searches with the websites Benchling (https://www.benchling.com), Deskgen (https://www.deskgen.com), CHOPCHOP (http://chopchop.cbu.uib.no), and CRISPRdirect (https://crispr.dbcls.jp). Three different pairs of annealed oligonucleotides encoding the gRNA sequences (vector I: 5'-CACCGCCGTGGCAACCCTGTGCTA-3' and 5'-AAACTAGCACAGGGTTGCCACGGC-3'; vector II: 5'-CACCGCTGTGTGAGCGCTACGCCA-3' and 5'-AAACTGGCGTAGCGCTCACACAGC-3'; vector III: 5'-CACCCTGCCTACAACCGCTACTTC-3' and 5'-AAACGAAGTAGCGGTTGTAGGCAG-3') were each inserted into the BbsI site of the targeting plasmid pX459 (Addgene). Each targeting vector was transfected into MS-5 BM stromal cells. Two days after transfection, 6.0% (vector I), 11.1 % (vector II), 3.2% (vector III), and 3.6% (control) cells were GFP-positive. These cells were sorted using FACSAria II (BD), and single cells were cultured in 96-well plates with the medium containing 50% fresh complete medium and 50% MS-5 cell culture supernatant. ELISA and western blot analyses were performed as described in the text.
