# Supplementary material for: Matrix Gla protein maintains normal and malignant hematopoietic progenitor cells by interacting with bone morphogenetic protein-4
Source: Heliyon. 2020 Apr 12;6(4):e03743. doi: 10.1016/j.heliyon.2020.e03743 (PMC7160454; doi:10.1016/j.heliyon.2020.e03743)

Supplementary material for Fig. 2.
Full non-adjusted images for Fig. 2B are shown.

Figure 2B

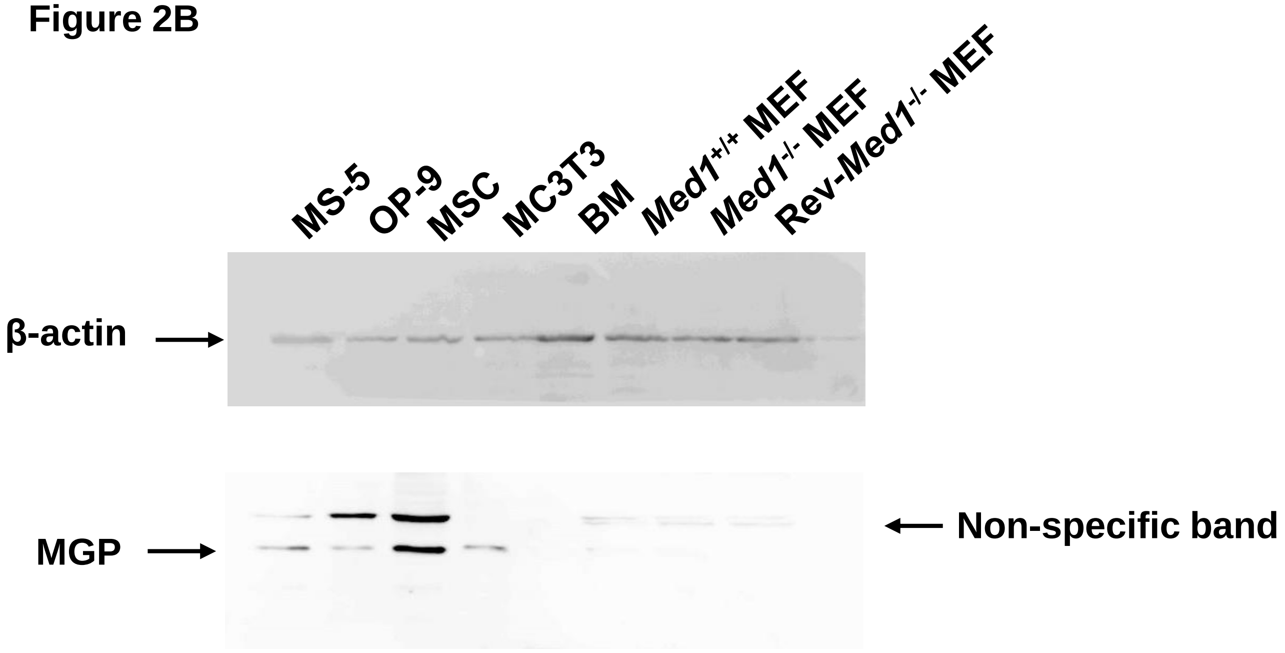

Supplementary material for Fig. 7.
Full non-adjusted images for Fig. 7A, 7B and 7D are shown.

Figure 7A

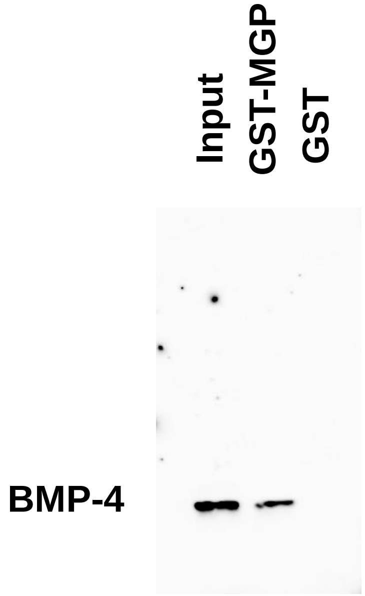

Figure 7B

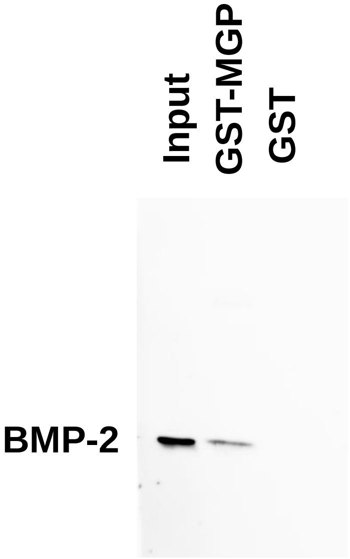

Figure 7D

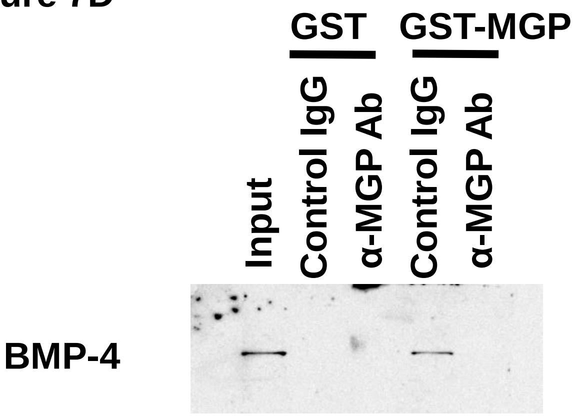

Supplementary material for Supplementary Fig. 1.
Full non-adjusted images for Supplementary Fig. 1C are shown.

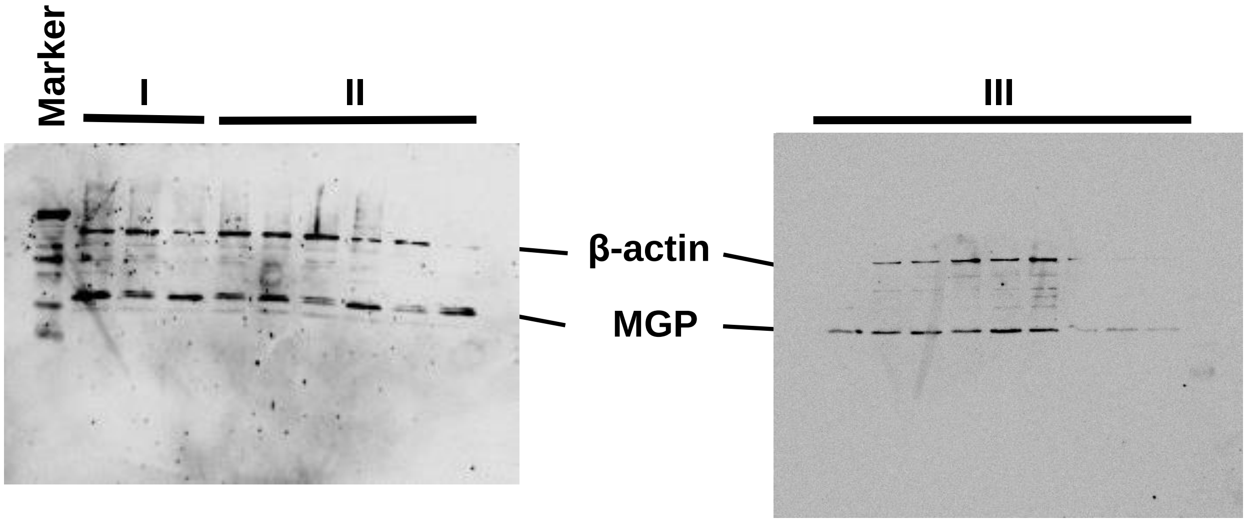

Supplement: Supplementary Material [file mmc2.pdf]
